# Severity, uncertainty, social support and coping style of parents who have children with epilepsy: a structural equation model

**DOI:** 10.3389/fneur.2025.1575628

**Published:** 2025-06-04

**Authors:** Miao Zhang, Liyun Lei, Dan Yao, Yongai Zhang

**Affiliations:** ^1^School of Nursing and Rehabilitation, Xi'an Medical University, Xi'an, China; ^2^Xi’an Children’s Hospital, Xi'an, China

**Keywords:** epilepsy, illness uncertainty, social support, active coping, parent

## Abstract

**Aim:**

To examine four variables in the model of perceived uncertainty in illness in northwestern China; to explore the relationship between severity, social support, illness uncertainty, and active coping in parents of children with epilepsy.

**Design:**

A cross-sectional study design.

**Reporting method:**

The STROBE checklist was used to ensure the rigor in this study.

**Method:**

This study recruits parents of children with epilepsy from a tertiary children’s hospital using convenience sampling between January and November 2024. Eligible participants completed questionnaires via an online platform (https://www.wjx.cn/) by scanning the QR code. Structural equation modeling and mediated effects serve as the methods for data analysis.

**Result:**

This study surveyed 492 parents, including 192 males (39.0%) and 300 females (61.0%). The corrected model achieved an acceptable model fit: (χ^2^ = 89.104 (*p* < 0.001); df = 59; χ^2^/df = 1.510; RMSEA = 0.043; CFI = 0.960; TLI = 0.941; IFI = 0.969). Severity positively predicted illness uncertainty (*β* = 0.105, *p* < 0.05). Social support negatively predicted illness uncertainty (*β* = −0.111, *p* < 0.05) and positively predicted active coping (*β* = 0.583, *p* < 0.001). Illness uncertainty negatively predicted active coping (*β* = −0.075, *p* < 0.05). Social support had a direct positive effect on active coping (*β* = 0.550, *p* < 0.01), and social support had an indirect negative effect on active coping through uncertainty (*β* = −0.012, *p* < 0.001).

**Conclusion:**

Illness uncertainty partially mediated the relationship between social support and active coping. However, we did not confirm a relationship between illness severity and active coping in this study.

## 1 Introduction

Epilepsy has become one of the most common neurological disorders, with approximately 50 million people living with epilepsy globally. It is estimated that 0.5–1% of children are diagnosed with epilepsy, and 80% of these children are in developing countries (1). The prevalence of epilepsy in children under the age of 19 years is approximately 50/100,000. Seizures are characterized by their repetitive, transient, stereotypical, and sudden nature (2). The unpredictability of seizures can be challenging for parents of epilepsy patients, as they may find themselves unable to anticipate the timing of the subsequent seizure and prepare for it in advance (3). It has been suggested that unpredictability may potentially contribute to psychological challenges, such as anxiety, stress, and depression, among parents (4, 5). Furthermore, studies have shown that parents of children with epilepsy tend to experience higher levels of anxiety, depression, poor sleep quality, and fatigue compared to parents of children without epilepsy (5–7). Mothers of children with epilepsy have been observed to experience a higher prevalence of post-traumatic stress disorder (PTSD) compared to fathers, and mothers are more prone to depression (8). Uncertainty worsens these feelings and influences long-term decision-making (6, 9).

Illness uncertainty can be understood as a cognitive state that arises when an illness-related event cannot be clearly defined, categorized, or predicted (10). According to Mishel (11), uncertainty can encompass various aspects, including ambiguity regarding the state of the illness, complexity surrounding treatment options, a lack of clarity on the illness’s severity, and unpredictability in the course of the illness. It is important to recognize that uncertainty is something that parents face on a daily basis as they manage their child’s illness. Parental uncertainty about their child’s diagnosis has been reported, especially in the early stages of diagnosis when parents need available information about etiology and comorbidities (12). The uncertainty experienced by parents of children with epilepsy often centers on managing convulsive seizures, including the anticipation of when and where the next one might occur, the severity of symptoms, medications, surgery, the efficacy of treatment, the prevention of injuries, the prognosis, and the reality that even if the doctor has informed them that their child will not grow up without convulsive seizures, this does not necessarily alleviate their uncertainty (6, 13–15). It has been observed that the uncertainty surrounding epilepsy may be distinctive from other disorders. Some parents shared that they often feel more uncertain during the nighttime hours, which can sometimes lead to fatigue (16). It is also important to acknowledge that parents of children with epilepsy may face challenges in acquiring comprehensive knowledge about epilepsy, formulating positive attitudes, and developing the necessary skills for effective caregiving practices (17, 18). While caring for their child, parents of children with epilepsy frequently face uncertainty when their child develops a new behavior. They must consider whether these changes indicate a new epileptic symptom or a side effect of medication, which can create a cycle of uncertainty (15). This uncertainty may persist even as the child transitions to adulthood. Parents encounter challenges in understanding waiting lists, health care payment systems, and where to access treatment (19). Social support has been identified as crucial in reducing uncertainty about the disease. Several studies have addressed this gap by emphasizing the importance of support from family, friends, coworkers, peer caregivers, and the healthcare team (20). In the context of children with epilepsy, the relationship between parents and medical staff follows a gradual process of trust-building, autonomy, and doubt (21). In a close, interactive patient-parent relationship, the parent can provide as much support, guidance, or comfort as possible during the care of the child (21). An RCT study validated the perceived level of nurse support for parents of children with adolescent epilepsy from an educational program based on individual and family self-management theory (22).

To the best of our knowledge, several studies have been conducted to explore the needs and experiences of parents of children with epilepsy using qualitative research methods (23–25). Research has shown that parents consistently experience uncertainty in caring for their child, parents learn how to manage uncertainty, parents recognize the need for peer support and health care providers in the community to alleviate uncertainty, worry and social isolation, and access to information (13), which can also change their coping manner or coping skills during the management of their child’s illness (4). It has been suggested that illness uncertainty in parents of children with epilepsy negatively impact their coping styles and access to social support (26, 27). However, it’s important to note that access to social support also carries potential risks and complications, such as the social stigma associated with disclosing a child’s diagnosis to others (28). There is some evidence that proactive communication and information-seeking can alleviate uncertainty about the disease in parents of children with epilepsy (7). Parent–child relationships, family functioning, and parental coping styles are more negative in children with epilepsy (6). Studies observe that girls receive more support from their parents than boys, which is particularly crucial during adolescence (29). It has been shown that the perceived social support of parents of children with epilepsy is inversely proportional to their levels of anxiety and stress (30). While the majority of existing research has focused on the experiences, needs, anxiety, depression, and uncertainty of parents of children with epilepsy (5, 14, 31), fewer studies have explored the development of a structural equation model to understand illness uncertainty. It is imperative to clarify the essential variables in order to implement interventions aimed at reducing uncertainty among parents and enhancing their quality of life (31).

### 1.1 Hypothesized model

**Figure 1:**
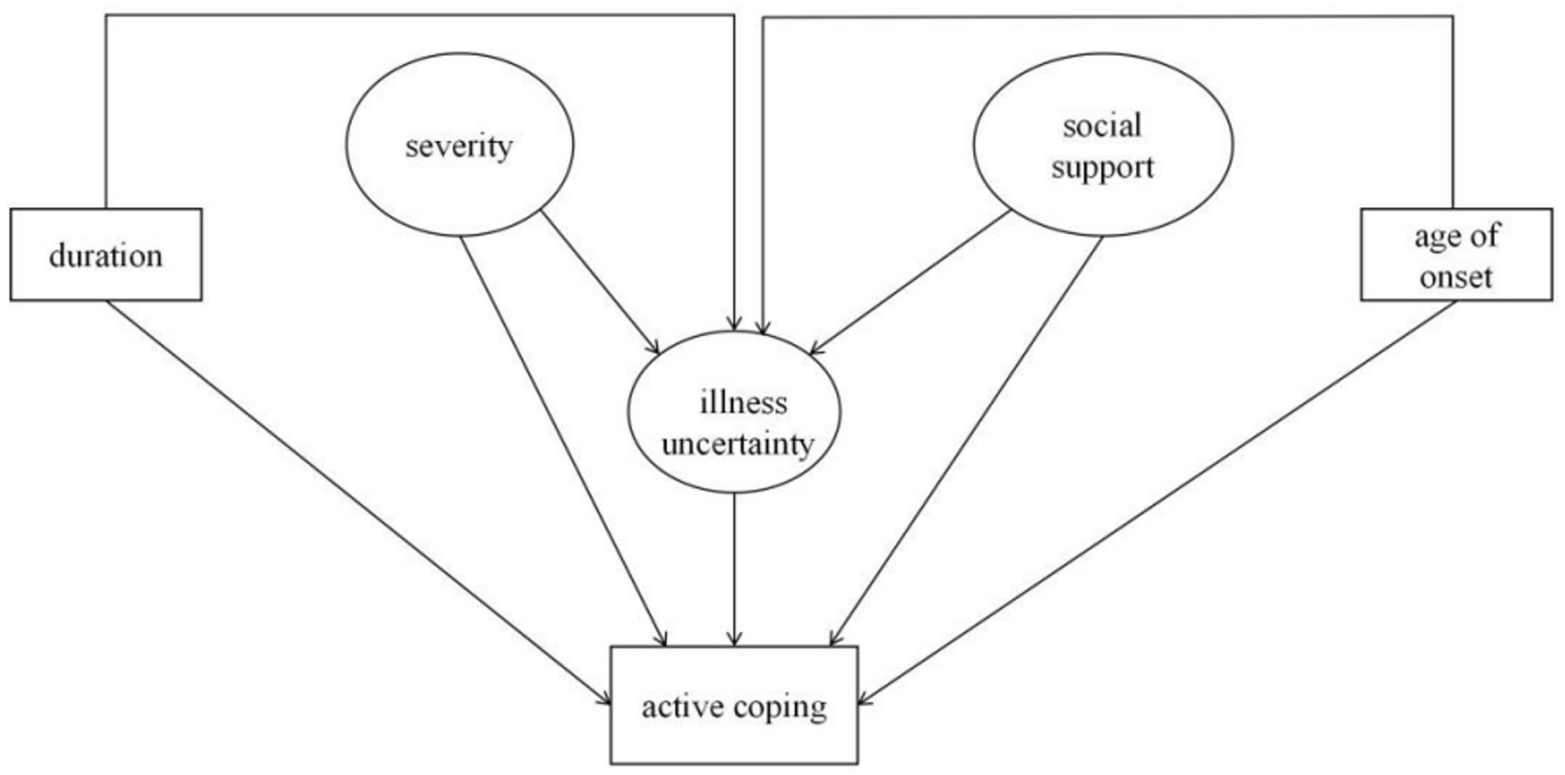
Hypothesized conceptual model.

A hypothetical model was constructed based on the Model of Perceived Uncertainty in Illness and the Reconceptualized Uncertainty in Illness, which explains how patients cognitively process illness-related stimuli and construct the meaning of these events. The original uncertainty theory pertains to acute illness, while the reconceptualized theory pertains to the continual uncertainty experienced in chronic illness. The model includes stimuli, illness uncertainty, structure providers, and coping strategies. In this study, we used epilepsy severity as a stimulus and social support as a provider. Both severity and social support were independent variables, while uncertainty was a mediation variable, and active coping functioned as a dependent indicator (see Figure 1). We controlled for the duration of the illness and the child’s age of onset based on a previous reference (32).

## 2 Methods

### 2.1 Aim

This study aimed to construct a structural equation model by using a cross-sectional survey with the severity of epilepsy, social support, illness uncertainty, and active coping as variables. A hypothesized conceptual model was shown in Figure 1.

Hypothesis 1: Severity positively affects illness uncertainty of parents and negatively affects active coping of parents.

Hypothesis 2: Social support negatively affects illness uncertainty of parents and positively affects active coping of parents.

Hypothesis 3: Illness uncertainty negatively affects active coping of parents.

Hypothesis 4: Illness uncertainty mediates the association between severity and active coping.

Hypothesis 5: Illness uncertainty mediates the association between social support and active coping.

### 2.2 Design and setting

The study was conducted using a cross-sectional study design at two neurology units in a children’s hospital in Xi’an, Shanxi province. This hospital (the name of this hospital has been deleted for anonymized review) is the largest tertiary, teaching, and specialized hospital for children in northwestern China.

### 2.3 Participants and sampling

We utilized convenience sampling, selecting parents of children who have been diagnosed with epilepsy for the study.

Parents were eligible if they were:

1. Their child was diagnosed with epilepsy;
2. Aged ≥18 years;
3. They were their child’s caregiver and lived with their child;
4. Either mother or father would be included if they were both at present;
5. Informed consent for participation.

Parents were excluded if they were suffering from cognitive impairment or mental illness or were unable to understand Chinese (33).

From January to November of 2024, we had the opportunity to collect data via an online survey. The researcher (first author) contacted the nursing department administrator and the head nurse of the Department of Neurology to request access to the patient’s medical records. This was done to identify participants who met the inclusion criteria. The first author contacted parents, and research nurses were trained to complete the questionnaire by scanning the QR code. The respondents completed the questionnaires on a time scale ranging from 3.85 to 42.4 min, with a mean of 14.52 min. A total of 550 parents of children with epilepsy met the inclusion criteria, and 503 of them agreed to complete the questionnaire. We meticulously screened the questionnaires for validity and excluded those deemed invalid from the analysis. We applied this exclusion criterion if the total response time was less than 128 s (equivalent to approximately 2 s per question for 64 questions) or if consecutive consistent responses comprised half or more of the questionnaire’s length (*n* = 11) (34, 35). In total, we evaluated 492 questionnaires. The sample size was determined using the *A-Priori* Sample Size for structural equation models software (36, 37), which indicated a minimum of 296 participants, accounting for an effect size of 0.2 (38, 39), a power of 0.2, a power of 0.80, a significance level of 0.05, 3 latent variables, and 12 observed variables. Although there is a referable effect size of 0.31 in previous studies (40), we chose a smaller effect size to ensure a larger sample size to test the model.

### 2.4 Measurements

#### 2.4.1 Demographics

The demographic characteristics included the child’s information (age, gender, duration of epilepsy (year), comorbidity, siblings, ketogenic diet) and the parents’ information (relationship with the child, age, marriage, education, occupation, family income/month, religion, and medical insurance).

#### 2.4.2 Epilepsy severity

The severity of the child’s epilepsy was assessed by the type of seizure (1 to 3), the frequency of seizures (0 to 3), and the number of antiepileptic drugs (AEDs) used (0 to 3), with a total score of 1 to 9 (41, 42). Data on the severity of the child’s disease were obtained from medical records. Seizure type: generalized tonic–clonic seizures were scored as 3, focal seizures were scored as 2, and absences were scored as 1. Seizure frequency: 3 for weekly or daily seizures, 2 for monthly seizures, 1 for one or twice per year, and 0 for no seizures in the last year. The number of AEDs: no medications 0, 1 medication 1, polytherapy with two AEDs 2, and polytherapy with three or more AEDs 3. A score of 1–5 was considered low epilepsy severity, and ≥6 as high epilepsy severity (41, 42).

#### 2.4.3 The Chinese version of the parent perception of uncertainty scale (PPUS)

We evaluated uncertainty using the Chinese version of the Parent Perception of Uncertainty Scale (PPUS). Mishel originally developed this scale in 1983 to assess parents’ response to their child’s illness and hospitalization (43), exhibiting high internal consistency with a Cronbach’s alpha coefficient of 0.91 (43). Mai et al. subsequently translated and adapted the scale in 2013 to assess uncertainty in parents of hospitalized children (44). The scale comprises four subscales: lack of clarity (8 items), multiattributed ambiguity (11 items), lack of information (5 items) and unpredictability (4 items), with a Cronbach’s alpha of 0.84. Respondents rate each item on a 5-point Likert scale, ranging from 1 (strongly disagree) to 5 (strongly agree), with total scores ranging from 28 to 140. Nine items (6, 9, 11, 19, 23, 25 ~ 28) are reverse scored. Higher scores denote greater levels of illness uncertainty. In the present study, the Cronbach’s alpha for the total scale was 0.850.

#### 2.4.4 Simplified coping style questionnaire (SCSQ)

Xie et al. (45) initially designed the scale in 1998 and demonstrated a Cronbach’s alpha of 0.89. He developed it to gauge an individual’s coping style. The scale includes 20 items and uses a 4-point Likert scale, with 0 signifying “not taking” and 3 indicating “often taking.” The active coping consists of 1st to 12th items and the passive coping consists of 13th to 20th items. The higher the active coping score, the lower the psychological problems or symptoms. For this study, only items related to active coping subscale were selected. In the current study, the Cronbach’s alpha for the subscale was 0.899.

#### 2.4.5 Social support rating scale (SSRS)

Xiao (46) developed the instrument in 1994 to quantify the extent of an individual’s social support. It has demonstrated robust reliability, boasting a Cronbach’s alpha of 0.89 ~ 0.94. It contains 10 items across three dimensions—subjective support (4 items), objective support (3 items) and utilization of support (3 items)—the scale is rated on a 4-point Likert scale or multiple scale. The total score spans from 12 to 66, with higher scores indicating higher levels of social support. In this study, the Cronbach’s alpha for SSRS was 0.746.

### 2.5 Ethical considerations

Before its implementation, the Ethics Committee of Xi’an Medical University (XYLS2024133) approved this study, and the study questionnaire did not include any private information (name, ID number, bed number). All data collected were used exclusively for academic research purposes. Furthermore, the consent of the study participants was obtained prior to the distribution of the questionnaire, and they were informed that their participation was voluntary and that their decision to participate or to withdraw from the study at any point would not have any adverse effect on the treatment and care of their children.

### 2.6 Data analysis

Data were analyzed using IBM SPSS, version 27.0. Descriptive statistics were used to summarize measurement data, while frequencies and percentages were reported for categorical data. Missing data were identified for the number of parents’ age (37), amounting to 8.1% missingness. To address this, a Missing Completely at Random (MCAR) test was conducted on the missing data, χ^2^(56) = 176.123, *p* = 0.151. The results suggested that the data were missing at random, which allowed for their imputation using multiple imputation techniques in SPSS. The automatic imputation method was selected, and the data were imputed five times to ensure robustness (47, 48). The absolute skewness and kurtosis of the variables (epilepsy severity, illness uncertainty, social support, and active coping) were less than 1 and less than 2, respectively, indicating that the data were normally distributed (49, 50). Pearson’s correlation coefficient was used to examine the relationships between severity, uncertainty, social support, and active coping.

The IBM SPSS Amos 24.0 was utilized for the structural equation model. The model fit indices including 1 < **χ**^2^/df < 3, the comparative fit index (CFI) and Tucker–Lewis index (TLI) ≥ 0.90, and root mean square error of approximation (RMSEA) < 0.08 indicate an accepted model fit (51). A *p*-value of ≤ 0.05 was considered statistically significant.

The hypothesized model was validated through a confirmatory factor analysis, which involved determining the observed indicators and corresponding factor loadings for each latent variable. Then, the model was fitted using the maximum likelihood estimation (MLE) to determine the relationships between the latent variables. If the model is poorly fitted, make corrections, including removing or modifying paths and adding errors and covariances. We performed a bootstrap analysis, 5,000 bootstrap samples, and 1,500 iterations to determine direct, indirect, and total effects. A 95% confidence interval (CI) that excludes zero was considered to indicate statistically significant.

## 3 Results

### 3.1 Sociodemographic characteristics of parents and their child

The demographic data is shown in Table 1. A comprehensive survey was conducted among 492 parents, with 192 males (39.0%) and 300 females (61.0%). The mean ages of the children and their parents were 5.85 ± 4.03 and 34.70 ± 4.29, respectively. The average duration of epilepsy (year) was found to be 1.64 ± 2.42. There were 120 (24.4%) children with comorbidities and 72 (14.6%) children on the ketogenic diet. The majority of the parents, 252 (51.2%), were married, 261 (53.0%) had high school education levels and below, and more than half of the parents, 300 (61.0%), reported monthly household incomes (yuan) of less than 5,000. All families had health insurance, with 216 (43.9%) reporting having urban resident basic medical insurance and 276 (56.1%) rural cooperative medical insurance. 432 (87.8%) parents reported no religion. 252 (51.2%) of parents were employed.

**Table 1:** Demographic characteristics of parents and their child (n = 492).

Characteristics	Frequency	Percentage (%)
Child		
Gender		
Female	180	36.6
Male	312	63.4
Age (year)		
≤5 (Median)	264	53.7
>5	228	46.3
Duration (year)		
≤1 (Median)	324	65.9
>1	168	34.1
Comorbidity		
Yes	120	24.4
No	372	75.6
Ketogenic diet		
Yes	72	14.6
No	420	85.4
Siblings		
Yes (healthy)	204	41.5
Yes (with illness)	12	2.4
No	276	56.1
Parents		
Relationship with the child		
Mother	300	61.0
Father	192	39.0
Age (year)		
≤33 (Median)	248	50.4
>33	244	49.6
Household structure		
Extended family	240	48.8
Nuclear family	192	39.0
Other	60	12.2
Marriage		
Married	252	51.2
Divorced	60	12.2
Other	180	36.6
Education		
High school or less	261	53.0
Bachelor’s degree or above	231	47.0
Household income/month (yuan)		
<5,000	300	61
5,000 ~ 10,000	156	31.7
10,001 ~ 15,000	24	4.9
>15,000	12	2.4
Insurance		
Urban resident basic medical insurance	216	43.9
Rural cooperative medical insurance	276	56.1
Religion		
Buddhism	36	7.3
Christianity	24	4.9
No religion	432	87.8
Employment		
Employed	252	51.2
Unemployed	60	12.2
Other	180	36.6

### 3.2 Correlation and descriptive analysis among the variables

Pearson correlation analysis revealed statistically significant correlations among the four variables (see Table 2). Specifically, there was a negative correlation between severity and social support (*p* < 0.05), a positive correlation between severity and active coping (*p* < 0.05), a positive correlation between social support and active coping (*p* < 0.01), a positive correlation between severity and uncertainty (*p* < 0.05), a negative correlation between social support and uncertainty (*p* < 0.05), and a negative correlation between uncertainty and active coping (*p* < 0.01).

**Table 2:** Descriptive and correlation of the variables.

Variables	Mean (±SD)	Correlation coefficient	1	2	3	4
1 Severity	1.67 (0.65)	r	1			
2 Social support	2.92 (0.57)	r	−0.073*	1		
3 Active coping	2.90 (0.52)	r	0.818*	0.392**	1	
4 Uncertainty	2.92 (0.27)	r	0.722*	−0.605*	−0.164**	1

r: Pearson coefficient. *p < 0.05. **p < 0.01.

### 3.3 The measurement model

A confirmatory factor analysis was completed to determine whether the observed variables would load onto the latent factor variable. The results showed that this model had a good fit (χ^2^ = 95.543 (*p* < 0.001); χ^2^/df = 1.676; RMSEA = 0.041; CFI = 0.971; TLI = 0.910; IFI = 0.977). All factor loadings were significant, but one item on the multiattributed ambiguity of the PPUS had a factor loading that was too low (0.33), but given that its AVE was 0.46 close to 0.5, we did not remove that item. In addition, the lack of information subscale had an AVE of 0.49, and the active coping dimension had an AVE of 0.45, which is close to 0.5 (see Table 3). The factor loadings for the items associated with the variables (severity, social support, uncertainty, and active coping) were all found to be significant (*p* < 0.05).

**Table 3:** Distribution and the results of confirmatory factor analysis.

Latent variables	Observed variables	Skewness	Kurtosis	Factor loadings of items		Average variance extracted	Composite reliability
Severity	Type	0.131	−0.910	0.60		0.43	0.69
	Frequency	0.016	−1.480	0.82			
	AEDs	0.370	−1.205	0.51			
Illness uncertainty (IU)	Multiattributed ambiguity	−0.373	0.768	IU3	0.66	0.46	0.90
				IU4	0.75		
				IU8	0.62		
				IU13	0.70		
				IU15	0.69		
				IU16	0.80		
				IU17	0.63		
				IU18	0.33		
				IU20	0.50		
				IU21	0.87		
				IU22	0.77		
	Lack of clarity	0.506	0.048	IU2	0.70	0.50	0.89
				IU5	0.62		
				IU6	0.71		
				IU7	0.85		
				IU9	0.64		
				IU10	0.57		
				IU14	0.66		
				IU28	0.84		
	Lack of information	0.445	0.675	IU1	0.57	0.49	0.82
				IU12	0.66		
				IU24	0.52		
				IU26	0.91		
				IU27	0.77		
	Unpredictability	0.032	−0.198	IU11	0.74	0.56	0.84
				IU19	0.70		
				IU23	0.82		
				IU25	0.73		
Social support (SS)	Subject support	0.166	−0.757	SS1	0.77	0.54	0.82
				SS3	0.81		
				SS4	0.72		
				SS5	0.62		
	Object support	−0.110	−0.386	SS2	0.48	0.67	0.85
				SS6	0.93		
				SS7	0.95		
	Support availability	0.370	−0.365	SS8	0.82	0.69	0.87
				SS9	0.85		
				SS10	0.83		
	Active coping (AC)	−0.012	−0.157	AC1	0.70	0.45	0.91
				AC2	0.71		
				AC3	0.64		
				AC4	0.80		
				AC5	0.66		
				AC6	0.78		
				AC7	0.65		
				AC8	0.61		
				AC9	0.70		
				AC10	0.58		
				AC11	0.62		
				AC12	0.53		

All factor loadings were significant at the 0.001 level (2-tailed). AEDs: antiepileptic drugs.

### 3.4 Structural equation model

**Figure 2:**
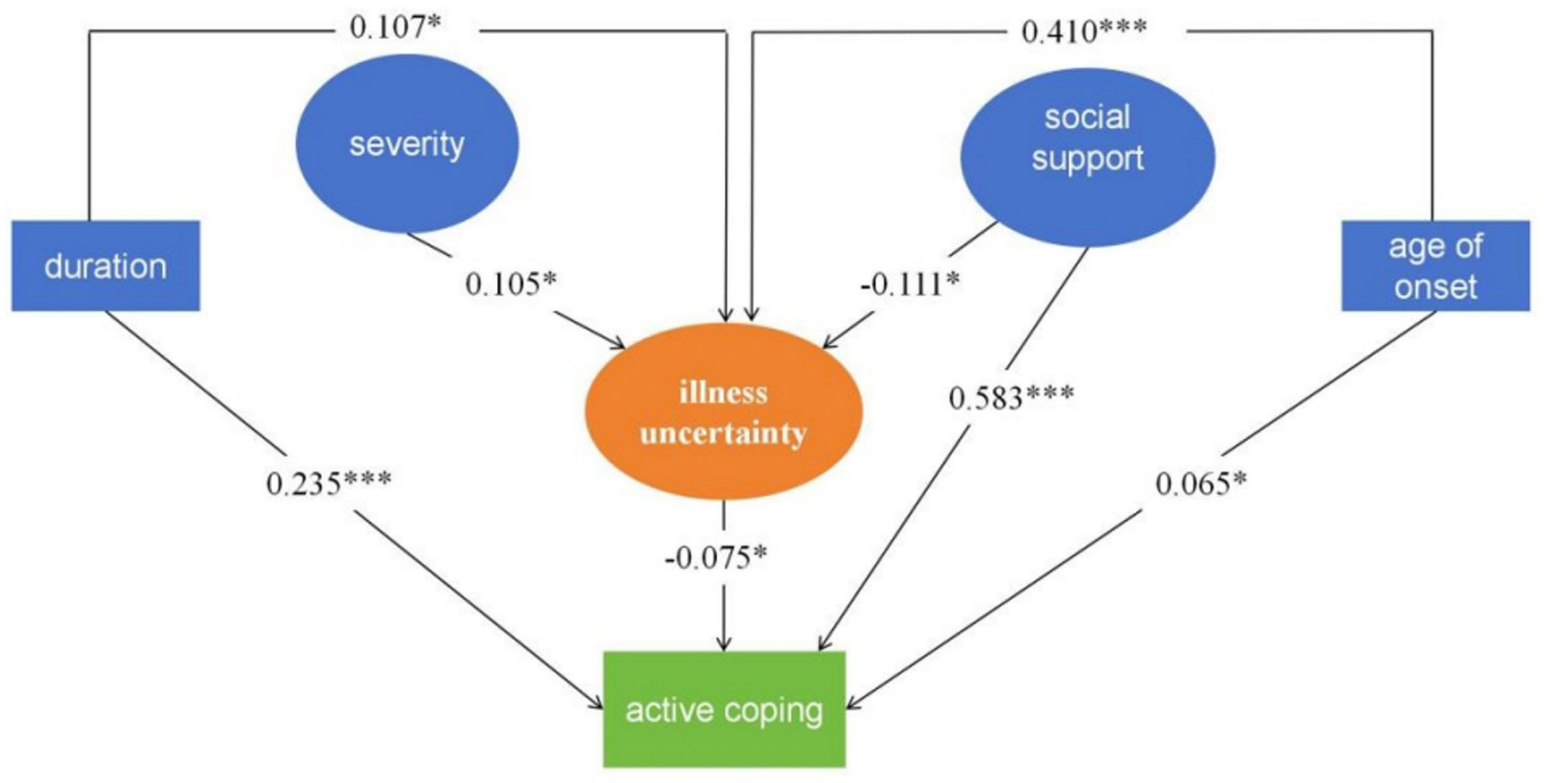
Final structural equation model. The boxes represent observed variables and the circles represent latent variables. *p < 0.05. **p < 0.01. ***p < 0.001.

The initial model is not acceptable according to the fit statistics (overall chi-square fit statistic is 205.664 with 57 degrees of freedom (df), *p*-value < 0.001, chi/df = 3.608; CFI = 0.824; TLI = 0.882; RMSEA = 0.07). We corrected the model by removing correlations that were not statistically significant (between severity and active coping). The corrected model had an acceptable model fit: (χ^2^ = 89.104 (*p* < 0.001); df = 59; χ^2^/df = 1.510; RMSEA = 0.043; CFI = 0.960; TLI = 0.941; IFI = 0.969). The duration of illness and age at onset were statistically significant in the model (see Figure 2). The relationship between severity and illness uncertainty of hypothesis 1 was confirmed as severity predicted illness uncertainty (*β* = 0.105, *p* < 0.05). Unexpectedly, severity did not positively predict active coping (*β* = 0.073, *p* = 0.367). Hypothesis 2 was confirmed as social support negatively predicted illness uncertainty (*β* = −0.111, *p* < 0.05) and positively predicted active coping (*β* = 0.583, *p* < 0.001). Hypothesis 3 was confirmed as illness uncertainty negatively predicted active coping (*β* = −0.075, *p* < 0.05).

### 3.5 Mediation analysis

Hypothesis 4 was not confirmed as severity predicted active coping through illness uncertainty (*β* = −0.217, *p* = 0.128). Hypothesis 5 was confirmed (see Table 4). Social support had a direct positive effect on active coping (*β* = 0.55, *p* < 0.01), and social support had an indirect negative effect on active coping through uncertainty (*β* = −0.012, *p* < 0.001).

**Table 4:** Mediating effects of uncertainty between social support and active coping.

Path	Effect	β	Standard error	95% CI	*p*
Social support → Active coping	Total effect	0.583	0.147	0.189, 0.762	***
Social support → Active coping	Direct effect	0.550	0.169	0.191, 0.801	**
Social support → Uncertainty → Active coping	Indirect effect	−0.012	0.090	−0.124, −0.004	***

**p < 0.01. ***p < 0.001.

## 4 Discussion

This study examines the model of perceived uncertainty in illness using a sample from a developing country. Gaining insight into these relationship is instrumental in guiding academic institutions and healthcare facilities in developing and implementing timely and tailored social support for parents of children with epilepsy.

Epilepsy severity in the present study sample was at a low level, lower than in previous studies (52, 53). Disease uncertainty was at about the same level as in other studies (27, 54), lower than in another study (55). Social support in this study was lower than in other studies (56, 57) and higher than the level of social support for fathers of children with epilepsy only (27). This discrepancy can be attributed to the fact that nearly half (43.9%) of the children in this study lived in extended families, which facilitated parents to seek social support from family members. Only 2 (0.4%) of the children in this study had no source of social support. The mean duration of illness for the children in this study was approximately 1.5 years, shorter than observed in other studies (27). This may lead to parents’ failure to seek social support within a shorter period. Secondly, it is possible that parental stigma in Asian culture (58) can influence parents to seek help. It has been demonstrated that negative emotions can diminish parents’ propensity to seek social support and adopt coping strategies (59). Furthermore, it has been observed that mothers may experience heightened anxiety compared to fathers, which could also contribute to a lower propensity to seek social support. Parents have shared that cooking classes can be a source of cooking skills and interpersonal support, which can help reduce feelings of isolation (20).

The present study sample demonstrated higher active coping in comparison with previous research (60), which may be attributable to the fact that the mean duration of illness in the present study was 1.5 years, and the parents may have already experienced a period of adjustment (61). This suggests that the parents may have adopted a positive coping approach. Higher levels of illness severity predict higher levels of illness uncertainty, and higher levels of social support may reduce illness uncertainty and enhance active coping. These results are consistent with existing theoretical frameworks and prior research findings (62–65). Furthermore, the study found that disease uncertainty negatively impacts active coping, which was a finding that aligns with prior research (66).

The shorter the duration of illness, the greater the sense of disease uncertainty, which is consistent with the results of Acuff and Jabson (67). This may be because parents acquire limited knowledge about treatment options, prognoses, and care for the illness over brief periods. Conversely, the duration of illness is positively correlated with active coping. This indicates that as the duration of illness increases, the likelihood of active coping by parents also rises, aligning with the findings of prior studies (61, 68). Furthermore, age at onset has been found to be positively associated with illness uncertainty, with older age being linked to more significant uncertainty among parents. This finding aligns with previous research (69). It is plausible that age is associated with more severe seizure type, with older children more prone to developing tonic–clonic seizures (70).

Furthermore, age at onset is a positive predictor of active coping. This suggests that parents are more likely to adopt active coping strategies as children get older, which aligns with previous research (71). This phenomenon may arise because, as children mature, they become more inclined to seek help or draw on peer support (72, 73). It is also possible that the diverse resources accessed by the child may be a driving factor in positive parental coping.

Although correlation analyses showed that illness severity positively and statistically significantly associated with active coping, which is consistent with Dean’s findings (74), yet inconsistent with some of them (62), it may have to be interpreted that mild-to-moderate illness severity may be able to act as a positive predictor of parental adoption of active coping styles. However, when the severity of the illness is high, it can lead to a reduction in parental hope, thereby lowering the probability of active coping. This hypothesis requires further validation through future research. In the final model, the causal relationship between the two variables was insignificant. This may be because the correlation analysis only examined two variables, while the structural equation modeling incorporated additional variables and control variables. In the mediation analysis, illness uncertainty partially mediated the relationship between social support and active coping. This suggests that social support directly and indirectly affects active coping. In other words, parents can enhance active coping by reducing illness uncertainty after receiving social support. They can also enhance active coping directly through social support.

Parents experience anxiety and panic at the shock of a diagnosis, which can exacerbate the sense of uncertainty about the disease. Adolescents with epilepsy and their parents experience uncertainty about whether to adapt or not to adapt during their child’s treatment (75). This ongoing uncertainty affects both the disease itself and future life trajectories, schooling, shifting adaptations during adolescence, adulthood, and subsequent partner relationships that may be altered. In addition to informing parents of the diagnosis, investigations, prognosis, and medications, healthcare professionals should encourage parents to communicate with other parents of children with the disease and increase their access to support. Studies have indicated that parents often lack information regarding how to handle their children’s reluctance to disclose their medical condition to others (76). It has been demonstrated that among pediatricians, there is still a lack of knowledge about epilepsy-related issues among pediatric providers other than neurologists (77). Discharge letters can play a supportive role in helping parents navigate this period of uncertainty (78). Communities, schools, and medical institutions can play a role in raising awareness by hosting public lectures to educate the public about epilepsy to reduce the stigma associated with it, which may be a “secondary wound” for parents.

In this study, a sample from a large tertiary children’s hospital in northwestern China was used to validate the model of uncertainty theory among parents of children with epilepsy. However, the variables included in this study were limited, and a more comprehensive set of variables, such as adaptation, should be considered in future stages. Personalized support strategies tailored to different personalities (79) support strategies should be considered for parents with different personalities, as some parents are not good at seeking support and should be provided with regular, diversified, and more accessible sources of information and online and offline interventions, such as ward corridors, lists of available support avenues, and recommended readings or materials. Future research could examine the differential effects of various support types (emotional, instrumental, informational) on the positive coping strategies of parents with pediatric patients. The program is designed for adolescents with epilepsy and their parents. Training for adolescents with epilepsy and their parents should enhance their knowledge and self-efficacy in disease management and enable them to remain in the ‘adjustment’ phase for extended periods (21, 80). Online education programs based on individual and family self-management theory enhanced parents’ perceived nurse-support level (22). Providers can set up interventions to reframe parents’ hopes for a cure (79). A notable concern among parents of children with early-onset epilepsy is the disease trajectory, followed by concerns regarding seizure control and medication side effects (81).

### 4.1 Limitations

Convenience sampling was utilized for participant selection, which may introduce bias and compromise the sample’s representativeness. The study’s scope was confined to a hospital within Xi’an City, Shaanxi Province, potentially limiting the generalizability of the findings to broader populations. The cross-sectional design of the research imposes constraints on establishing causal relationships between the variables.

## 5 Conclusion

This study validated the theory of illness uncertainty in a population in northwestern China, explored the relationship between illness severity, illness uncertainty, social support, and active coping, and validated structural equation modeling between the variables with duration of illness and age at onset as control variables. Severity positively predicted illness uncertainty. Social support negatively predicted illness uncertainty and positively predicted active coping. Illness uncertainty negatively predicted active coping. Illness uncertainty partially mediated the relationship between social support and active coping. Timely and comprehensive support can play a vital role in alleviating the uncertainty, especially in the early phase of treatment. Future research could explore parental barriers to seeking social support and attempt to include additional variables, including uncertainty appraisal and psychological adjustment.

## Data Availability

The original contributions presented in the study are included in the article/supplementary material, further inquiries can be directed to the corresponding author.
