# Correction: Severity, uncertainty, social support and coping style of parents who have children with epilepsy: a structural equation model

**DOI:** 10.3389/fneur.2025.1652831

**Published:** 2025-07-08

**Authors:** Miao Zhang, Liyun Lei, Dan Yao, Yongai Zhang

**Affiliations:** ^1^School of Nursing and Rehabilitation, Xi'an Medical University, Xi'an, China; ^2^Xi'an Children's Hospital, Xi'an, China

**Keywords:** epilepsy, illness uncertainty, social support, active coping, parent

In the published article, there was an error in the **Funding** statement. The new statement appears below:

